# School nurses’ attitudes and experiences regarding the human papillomavirus vaccination programme in Sweden: a population-based survey

**DOI:** 10.1186/1471-2458-14-540

**Published:** 2014-05-31

**Authors:** Maria Grandahl, Tanja Tydén, Andreas Rosenblad, Marie Oscarsson, Tryggve Nevéus, Christina Stenhammar

**Affiliations:** 1 Department of Public Health and Caring Sciences, Uppsala University, Box 564, SE-751 22 Uppsala, Sweden; 2 Centre for Clinical Research Vasteras, Uppsala University, Central Hospital, Vasteras, Sweden; 3 School of Health and Caring Sciences, Linnaeus University, Kalmar, Sweden; 4 Department of Women’s and Children’s Health, Uppsala University, Uppsala, Sweden

**Keywords:** Attitude, Experience, Human papillomavirus, School health, School nurse, Vaccination

## Abstract

**Background:**

Sweden introduced a school-based human papillomavirus (HPV) vaccination programme in 2012, and school nurses are responsible for managing the vaccinations. The aim of the present study was to investigate the attitudes and experiences of school nurses regarding the school-based HPV vaccination programme 1 year after its implementation.

**Methods:**

Data were collected using a web-based questionnaire in the spring of 2013, and 83.1% (851/1024) of nurses responded.

**Results:**

There were strong associations between the nurses’ education about the HPV vaccine and their perceived knowledge about the vaccine and a favourable attitude towards vaccination (both *p* < 0.001). School nurses who received a high level of education were more likely to have a positive attitude to HPV vaccination compared with nurses with little education about HPV vaccination (adjusted odds ratio [OR] = 9.8; 95% confidence interval [CI]: 3.797–25.132). Nurses with high perceived knowledge were more likely to have a positive attitude compared with those with a low level of perceived knowledge (OR = 2.5; 95% CI: 1.299–4.955). If financial support from the government was used to fund an additional school nurse, nurses were more likely to have a positive attitude than if the financial support was not used to cover the extra expenses incurred by the HPV vaccination (OR = 2.1; 95% CI: 1.051–4.010). The majority, 648 (76.1%), had been contacted by parents with questions about the vaccine, mostly related to adverse effects. In addition, 570 (66.9%) stated that they had experienced difficulties with the vaccinations, and 337 (59.1%) of these considered the task to be time-consuming.

**Conclusions:**

A high level of education and perceived good knowledge about HPV are associated with a positive attitude of school nurses to the HPV vaccination programme. Thus, nurses require adequate knowledge, education, skills and time to address the questions and concerns of parents, as well as providing information about HPV. Strategic financial support is required because HPV vaccination is a complex and time-consuming task.

## Background

Human papillomavirus (HPV) vaccination programmes for girls aged 9–14 years have been implemented in many countries worldwide [1,2]. Implementation strategies vary between countries because of different healthcare policies. In the United States as well as in several European countries, the vaccine is delivered in a primary care setting or health centres. In other countries, such as Australia, Canada and the United Kingdom, vaccination is performed in schools [3]. The vaccination uptake rate for three doses differs between countries, but it is in general highest in locations and countries where school-based vaccination programmes have been implemented [3,4], varying from about 70% in Australia [5] to 84% in the United Kingdom [6].

In 2012, the quadrivalent HPV vaccine was introduced in a Swedish school-based vaccination programme for girls aged 10–12 years. The first-year coverage for at least one dose was 79% [7]. All school-based vaccinations are free of charge, but parents must provide consent [8]. School nurses are responsible for managing all aspects of the vaccinations, including the logistics, providing information to parents and girls, and administration of the vaccine. The information distributed to parents is standardised by the National Board of Health and Welfare, which ensures that all parents receive equal and uniform information [8]. It is recommended that school nurses work in pairs during vaccination to facilitate the management of adverse reactions. After receiving the injected vaccine, the girls are observed for at least 15 minutes. As school nurses generally work alone, an extra nurse is required during vaccinations, and this is arranged by employing an additional nurse or by school nurses providing reciprocal cover with a colleague in a school nearby. To compensate the School Health Services for this increased workload, the Swedish Government has provided extra financial support to the municipalities. The principal of each school is responsible for the distribution of this funding.

The perceptions of Swedish school nurses regarding the HPV vaccination programme were explored during focus group interviews before its implementation [9]. The school nurses considered that the vaccine would be beneficial, but they had doubts about its safety. They were also concerned about their workload, as well as how they would inform about HPV and obtain informed consent. In general, the school nurses supported the implementation, and they believed that it would decrease inequalities between families of different socioeconomic status [9]. In the United Kingdom, where the school-based vaccination programme started in 2008, school nurses believed that HPV vaccination was a way to minimise health inequalities, although the procedure gave them less time for other crucial responsibilities, such as supporting vulnerable pupils [10,11].

An efficient vaccination programme is important to achieve high coverage, and school nurses play a key role in the success of school-based HPV vaccination programmes. Thus, the aim of the present study was to investigate the attitudes and experiences of school nurses regarding the school-based HPV vaccination programme 1 year after its implementation in Sweden.

### Hypothesis

The hypothesis was that there would be associations between school nurses (i) perceived knowledge of HPV vaccinations, (ii) the education they received about HPV, and (iii) the financial support given to the School Health Services, on one hand, and the attitudes of school nurses to the HPV vaccination programme on the other hand.

## Methods

### Design

A cross-sectional web-based survey was carried out in Sweden between March and May 2013. The school nurses eligible for inclusion were those who participated in the school-based HPV vaccination programme. At the time of the study, there were 2438 registered school nurses in Sweden, and 1024 worked in the 5th and 6th grades (children aged 10–12 years), which were the ages included in the school-based HPV vaccination programme.

### Procedure

Nurses were recruited via the official website of the Swedish Association of School Nurses. One of the researchers (CS) sent an email explaining the study’s aim and procedures to all heads of the School Health Services, as well as a request to inform the school nurses in each municipality about the study and to invite them to participate. More information was available on the website, including a statement that participation in the study was voluntary and confidential. Those who agreed to participate were asked to complete the web-based questionnaire. Reminders were sent by email to the heads of the School Health Services on two occasions, 2 and 4 weeks after the first invitation. The study was approved by the Regional Ethical Review Board in Uppsala, Sweden.

### Questionnaire

The questionnaire was based on previous studies [9,12] and clinical experience. The first part captured background demographic data, with questions about age, sex, years of experience as a school nurse, responsible school authority (public or private), which county they worked in, and also the geographical area (rural, semi-urban or urban). The main part of the questionnaire comprised questions about the education and support given to nurses before programme implementation, information provided to families by the school nurse and the school nurse’s perceived knowledge of, and attitude towards, HPV vaccination. The questions had multiple choice alternatives, which were rated using four-point scales that ranged from “strongly agree” to “strongly disagree”. The validity of the questionnaire [13] was tested using a group of 10 school nurses, who were asked to assess the relevance of the questions and to indicate whether they experienced any difficulties or ambiguity during its completion. Some revisions were made, and the final version comprised 26 questions, including two open-ended questions that asked participants to state the most common question asked by parents and to describe their perceived difficulties with HPV vaccinations. These open-ended questions were analysed by systematically organising and categorising the answers. The qualitative components of this study adheres to the RATS guidelines for reporting qualitative studies.

The outcome in the present study, attitude towards HPV vaccination, was based on two questions: (i) whether the school nurses agreed that the introduction of HPV vaccinations into the general childhood vaccination programme was appropriate; and (ii) whether they agreed that school nurses should be responsible for these vaccinations. The answers were scored as “strongly disagree” = 0, “partially disagree” = 1, “partially agree” = 2, and “strongly agree” = 3. The scores from the two questions were added, resulting in a total score of 0–6, categorised as “positive attitude” = 6, “ambivalent attitude” = 4–5, and “negative attitude” = 0–3.

Of the three main predictors in the present study, perceived knowledge of HPV vaccinations was based on two questions about whether the school nurses had good knowledge about HPV to inform (i) the girls and (ii) their parents or guardians before the vaccination. These were scored in the same way as for attitude towards HPV vaccination, with the total score categorised as “high perceived knowledge” = 6, “ambivalent about perceived knowledge” = 4–5, and “low perceived knowledge” = 0–3. Nurses’ education about HPV vaccination was based on three questions about whether they had received and considered information from (i) governmental agencies and (ii) pharmaceutical companies, and (iii) if they had knowledge about the campaigns against HPV. These questions were scored in the same way as for attitude towards HPV vaccination, resulting in a score of 0–9. Further, an extra point was added for each of three alternative levels of formal organised training (national, local, individual) they had received or if they had searched for information on their own. This resulted in a total score of 0–13 for education received, with the total score categorised as “high level of education” = 11–13, “medium level of education” = 8–10, and “low level of education” = 0–7. Financial support given to the School Health Services was categorised into three categories: (i) used to fund an additional school nurse; (ii) used for other purposes (to fund extra work time, continuous education, or equipment); and (iii) not used to cover extra expenses incurred by the HPV vaccinations.

### Statistical analyses

Categorical and ordinal data are presented as frequencies and percentages, n (%), and continuous data as means and standard deviations (SD). Correlations between ordinal variables were calculated using Spearman’s *ρ*. Univariate tests for differences between the three groups (“positive”, “ambivalent”, and “negative” attitude to HPV vaccination) were performed using Pearson’s χ^2^-test for categorical and ordinal variables, while analysis of variance was used for continuous variables. In analyses using the total score (0–6) of the variable attitude to HPV vaccination, differences in attitude between two categories were tested using the Mann–Whitney *U* test, while the Kruskal-Wallis test was used to test for differences between more than two categories.

Multinomial logistic regression analysis was used to study the magnitude of the association between the three main predictors and the outcome. In the regression models, “negative attitude” was used as the reference category for the outcome “attitude to HPV vaccination”. The regression analyses were performed in two steps. First, univariate logistic regression analyses were performed for each of the variables with *p* < 0.2 from the univariate tests for differences between the three groups “positive”, “ambivalent”, and “negative” attitude. Second, all variables with *p* < 0.2 in the univariate logistic regression analyses were then simultaneously used in multivariate logistic regression analysis to obtain the adjusted odds ratios (ORs) of the included variables for having a positive or ambivalent attitude to HPV vaccination compared with having a negative attitude. The results of the logistic regression analyses are presented as ORs with 95% confidence intervals (CIs) and accompanying *p*-values.

All statistical analyses were performed using SPSS Statistics version 20.0 (IBM Corp., Armonk, NY, USA). In all analyses, two-sided *p*-values <0.05 were considered statistically significant.

## Results

In total, 83.1% (851/1024) of the school nurses from all counties in Sweden completed the web-based questionnaire. The characteristics of the participating nurses, according to their attitudes to HPV vaccination, are presented in Table 1. There were statistically significant differences in attitudes to HPV vaccination related to perceived knowledge about HPV vaccination (*p* < 0.001) and education received about HPV (*p* < 0.001). Nurses were more likely to have a positive attitude to vaccination if they had more knowledge and education about vaccination.

**Table 1:** Characteristics of the participating school nurses (n = 851) according to attitude to human papillomavirus (HPV) vaccination

	**Attitude to HPV vaccination**			
**Variables**	**Positive (n = 446)**	**Ambivalen (n = 314)**	**Negative (n = 91)**	***p-*** **value**^**a**^
Age (years), mean (SD)	52.0 (7.7)	51.9 (7.2)	50.6 (7.5)	0.276
Years as a school nurse, mean (SD)	10.6 (7.9)	9.7 (6.6)	9.6 (5.9)	0.190
Sex				0.309^b^
−Female, n (%)	438 (52.1)	311 (37.0)	91 (10.8)	
−Male, n (%)	8 (72.7)	3 (27.3)	0 (0.0)	
Employment				0.364
−Public school, n (%)	411 (52.6)	290 (37.1)	80 (10.2)	
−Private school, n (%)	35 (50.0)	24 (34.3)	11 (15.7)	
Perceived knowledge				<0.001
−High	277 (60.1)	152 (33.0)	32 (6.9)	
−Ambivalent	120 (42.7)	120 (42.7)	41 (14.6)	
−Low	49 (45.0)	42 (38.5)	18 (16.5)	
Education received about HPV				<0.001
−High	122 (62.2)	67 (34.2)	7 (3.6)	
−Medium	290 (52.7)	200 (36.4)	60 (10.9)	
−Low	33 (31.7)	47 (45.2)	24 (23.1)	
Use of financial support				0.126
−Found an additional school nurse	108 (59.0)	63 (34.4)	12 (6.6)	
−Other	32 (51.6)	25 (40.3)	5 (8.1)	
−Not used to cover extra expenses	305 (50.4)	226 (37.4)	74 (12.2)	

^a^*p*-values from ANOVA and Pearson’s χ^2^-test for categorical and ordinal variables.

^b^Unreliable because of too few male school nurses included.

### Attitudes to HPV vaccination

In total, 446 nurses (52.4%) had a positive attitude to HPV vaccination, 314 (36.9%) had an ambivalent attitude, and 91 (10.7%) had a negative attitude (Table 2). The majority of nurses strongly or partially agreed that it was appropriate that HPV vaccinations were introduced into the general childhood vaccination programme and that school nurses should be responsible for the vaccinations. Most participants agreed, strongly or partially, that boys should also be offered the HPV vaccine in the school-based vaccination programme. One out of 10 believed that a provider other than the School Health Services, such as primary care, n = 74 (8.7%) or a vaccination agency n = 11 (1.3%), would be more appropriate.

**Table 2:** School nurse attitudes towards HPV vaccination (n = 851)

	**Strongly agree**	**Partially agree**	**Partially disagree**	**Strongly disagree**
	**n (%)**	**n (%)**	**n (%)**	**n (%)**
It is appropriate that HPV vaccination was introduced into the general childhood vaccination program.	544 (63.9)	239 (28.6)	54 (6.2)	11 (1.3)
The school nurse should be responsible for the HPV vaccinations.	517 (60.8)	239 (28.1)	68 (8.0)	27 (3.2)
Boys should also be offered the HPV vaccine in the school-based vaccination program.	352 (41.4)	341 (40.1)	113 (13.3)	45 (5.3)
HPV vaccination is more painful than other vaccinations.	232 (27.3)	302 (35.5)	172 (20.2)	145 (17.0)
Fear of needles is a problem.	198 (23.3)	375 (44.1)	176 (20.7)	102 (12.0)
Those who decline vaccination should be offered the vaccination later.	271 (31.8)	238 (28.0)	184 (21.6)	158 (18.6)
I have sufficient confidence in the authorities’ decisions about HPV vaccination.	393 (46.2)	356 (41.8)	81 (9.5)	21 (2.5)
HPV vaccination may result in decreased condom use.	54 (6.3)	377 (44.3)	257 (30.2)	163 (19.2)
HPV vaccination may result in an increased number of sexual partners.	31 (3.6)	167 (19.6)	335 (39.4)	318 (37.4)
HPV vaccination may improve the awareness of sexually transmitted infections.	199 (23.4)	527 (61.9)	95 (11.2)	30 (3.5)
HPV vaccination may reduce participation in Pap smear tests.	72 (8.5)	466 (54.8)	227 (26.7)	86 (10.1)

In addition, 574 school nurses (67.5%) considered that it was appropriate to vaccinate girls aged 10–12 years, whereas 238 (28.0%) thought that 13–14 years was a more suitable age, and 39 (4.5%) stated that the vaccine should be given at ≥ 15 years.

In their answers to the open-ended question, 570 of the participants (67%) reported difficulties with the HPV vaccinations, including some who had encountered more than one difficulty. We found that 276 of these school nurses (48.4%) had doubts about vaccination at such a young age, while 337 (59.1%) considered that the HPV vaccination programme was time-consuming, and 115 (20.2%) believed that their workload had increased. They believed that it would be easier to provide information about HPV infection if the girls were older and more sexually active and to motivate parents because most Swedish girls are not sexually active before the age of 15. Fears of needles and pain were highlighted by 148 nurses (26.0%), and 85 (14.9%) of the respondents reported logistical problems with the time intervals between the three doses. One school nurse expressed her difficulties as follows: *“The HPV vaccinations increase our workload, because they are very time-consuming and require a lot of planning. Therefore, it is challenging to find time for other preventive efforts”.*

### Received education and perceived knowledge about HPV

Table 3 examines the education the school nurses received about HPV vaccination and their perceived knowledge about it. Using total scores, a positive correlation between the nurses’ perceived knowledge about vaccination and a favourable attitude towards vaccination was found (*ρ* = 0.191; *p* < 0.001). There was also a positive correlation between the education received about HPV vaccination and a favourable attitude (*ρ* = 0.025; *p* < 0.001).

**Table 3:** School nurses’ perceived knowledge about HPV vaccination (n = 851)

	**Strongly agree**	**Partially agree**	**Partially disagree**	**Strongly disagree**
	**n (%)**	**n (%)**	**n (%)**	**n (%)**
I have received and considered the Government´s information about HPV.	661 (77.7)	136 (16.0)	54 (6.3)	0 (0.0)
I have received and considered the drug company’s information about HPV.	476 (55.9)	299 (35.1)	70 (8.2)	6 (0.7)
I have knowledge about the campaigns against HPV	461 (54.2)	367 (43.1)	23 (2.7)	0 (0.0)
I have good knowledge about HPV to inform parents.	481 (56.5)	296 (34.8)	61 (7.2)	13 (1.5)
I have good knowledge about HPV to inform girls.	529 (62.2)	226 (26.6)	96 (11.3)	0 (0.0)

In total, 534 (62.7%) of the respondents received formal organised training about HPV before the implementation at a local level, i.e., by their employer. In addition, 565 (66.4%) of the nurses obtained information themselves, 192 (22.6%) participated in formal training organised by the National Board of Health and Welfare or other national education programmes, and 109 (12.8%) of the nurses received individual organised training, e.g. by a school physician. However, 43 (5.1%) of the nurses received no formal organised education about HPV. The majority, 788 (92.6%), were content with both the Government’s information and the information provided by the drug companies.

### Information about HPV

Most of the school nurses, 828 (97.3%), sent standardised written information to the parents, and 175 (20.6%) also provided information at parental meetings. In addition, 290 of the school nurses (34.1%) provided information by email or telephone if they were contacted by the parents. We found that 466 (54.7%) informed the girls separately before the vaccination, whereas 262 (30.8%) informed both girls and boys together, and 123 (14.5%) did not inform the pupils verbally before the vaccination. The majority of the school nurses, 648 (76.1%), were contacted by parents who had questions about the vaccine. More than half of these questions, 355 (54.8%), were related to the adverse effects of the vaccine, while the other queries raised by parents were concerned with vaccination at such a young age (122 (18.8%)), and the long-term effects of the vaccine (85 (13.1%)). Moreover, 86 nurses (13.3%) were contacted by parents for advice about the vaccination. One school nurse illustrated a parent’s concerns as follows: *“Is the vaccine reliable, and what are the common side effects? Is it necessary to vaccinate a girl aged 10–12 or is it possible to wait until she is older?”.*

### Financial support from the government

No statistically significant differences in attitudes to HPV vaccination were observed overall in Table 1 for the different uses of the financial support from the government (*p* = 0.126). However, an analysis of the total score (0–6) for attitude to HPV vaccination revealed a statistically significant (*p =* 0.044) difference in attitude due to how the financial support from the Swedish Government had been used. The nurses who worked at schools that used the money to fund the costs of an additional school nurse had a significantly (*p =* 0.014) more favourable attitude towards HPV vaccination compared with those who worked at schools where the money was not used to compensate for the additional expenditure incurred by the vaccinations. The majority of the participants, 652 (76.6%), reported that as far as they knew, the financial support had not been used to cover the extra expenses incurred by HPV vaccinations. Less than a quarter, 199 (23.4%), had received financial support, 183 (21.5%) for an additional nurse, and 16 (1.9%) for extra working time.

### Multinomial logistic regression analyses

Tables 4 and 5 present the results from the univariate and multivariate, respectively, multinomial logistic regression analyses with attitude to HPV vaccination as outcome. The number of years as a school nurse, perceived knowledge about HPV vaccination, education about HPV vaccination and use of financial support from the government all had *p*-values <0.2 in the univariate tests for differences between the three groups “positive”, “ambivalent”, and “negative” attitude presented in Table 1. Thus, they were all included in separate univariate logistic regression analyses. All variables except years as a school nurse showed a statistically significant association with attitude to HPV vaccination, and were thus included simultaneously in the multivariate logistic regression.

**Table 4:** Univariate logistic regression with attitude to HPV vaccination as outcome

	**Attitude to HPV vaccination**			
	**Positive**^**a**^		**Ambivalent**^**a**^	
**Variable**	**OR (95% CI)**	***p*-value**	**OR (95% CI)**	***p*-value**
Years as a school nurse	1.020 (0.987–1.054)	0.237	1.003 (0.969–1.038)	0.869
Perceived knowledge				
−High	3.180 (1.655–6.107)	0.001	2.036 (1.040–3.983)	0.038
−Ambivalent	1.075 (0.563–2.052)	0.826	1.254 (0.650–2.418)	0.498
−Low	Ref.		Ref.	
Education received				
−High	12.675 (5.023–31.985)	<0.001	4.888 (1.946–12.275)	0.001
−Medium	3.515 (1.939–6.372)	<0.001	1.702 (0.962–3.011)	0.067
−Low	Ref.		Ref.	
Use of financial support				
−Found an additional school nurse	2.184 (1.141–4.176)	0.018	1.719 (0.878–3.363)	0.113
−Other	1.553 (0.585–4.122)	0.377	1.637 (0.605–4.430)	0.332
−Not used to cover extra expenses	Ref.		Ref.	

^a^Reference category: Negative attitude.

Unadjusted odds ratios (OR) and 95% confidence intervals (CIs).

**Table 5:** Multinomial logistic regression with attitude to HPV vaccination as outcome (adjusted ORs (95% CIs))

	**Attitude to HPV vaccination**			
	**Positive**^**a**^		**Ambivalent**^**a**^	
**Variable**	**OR (95% CI)**	***p*-value**	**OR (95% CI)**	***p*-value**
Perceived knowledge				
−High	2.537 (1.299–4.955)	0.006	1.758(0.889–3.474)	0.104
−Ambivalent	1.083 (0.558–2.101)	0.813	1.270 (0.654–2.465)	0.480
−Low	Ref.		Ref.	
Education received				
−High	9.769 (3.797–25.132)	<0.001	4.276 (1.676–10.907)	0.002
−Medium	3.154 (1.707–5.826)	<0.001	1.632 (0.910–2.924)	0.100
−Low	Ref.		Ref.	
Use of financial support				
−Found an additional school nurse	2.053 (1.051–4.010)	0.035	1.655 (0.837–3.272)	0.147
−Other	1.118 (0.410–3.046)	0.827	1.331 (0.484–3.657)	0.579
−Not used to cover extra expenses	Ref.		Ref.	

^a^Reference category: Negative attitude.

The results from the multivariate logistic regression model showed a strong positive association between education received and attitude to HPV vaccination. Thus, a school nurse with a high level of education about HPV vaccination was 9.8 times more likely to have a positive attitude to HPV vaccination compared with a nurse with a low level of received education (*p* < 0.001). Likewise, a school nurse with a medium level of received education was 3.2 times more likely to have a positive attitude, compared with a nurse with a low level of received education (*p* < 0.001). Also perceived knowledge about HPV vaccination had a positive association with attitude to HPV vaccination, with those having a high perceived knowledge being 2.5 times more likely to have a positive attitude compared with those with a low level of perceived knowledge (*p* = 0.006). Finally, if the financial support from the government was used to fund an additional school nurse, the school nurse was 2.1 times more likely to have a positive attitude, compared with nurses working in schools where financial support was not used to cover the extra expenses incurred by HPV vaccination (*p* = 0.035).

## Discussion

To the best of our knowledge, this is the first study to examine the attitudes and experiences of school nurses regarding a HPV vaccination programme at the population level.

School nurses have a key role in the success of the implementation of vaccination programmes [10,11]. Therefore it is crucial that they have adequate knowledge about HPV and skills to ensure that they feel comfortable with all the procedures related to the vaccinations. Adequate knowledge and appropriate ongoing education about HPV among healthcare professionals (HCPs) are necessary in order to promote vaccine coverage [14,15]. Thus, the nurses who had a positive attitude to the HPV vaccinations and were confident with their training were likely to be more at ease when providing information to parents and children, managing their concerns and obtaining informed consent. School nurses have a unique position in informing parents and addressing their questions and concerns (and clarifying any misunderstandings) regarding HPV vaccination [16,17]. Their communication skills regarding HPV vaccination are essential to foster parental acceptance and to promote public health [14]. Recommendations from HCPs are associated with better HPV vaccine uptake [15,18]. Thus, HCP education and knowledge about HPV as well as communication skills are important since the majority were contacted by parents who had additional questions about the vaccine, especially its adverse effects. The necessity for HCPs to discuss vaccine safety and effectiveness with parents was also noted in previous studies [17,19]. The safety profile of the HPV vaccine is well established, and no severe adverse effects have been found [20]. Thus, if this information is highlighted more clearly in the printed information provided to parents, some of their concerns may be alleviated. This would also make the task of obtaining informed consent easier for school nurses.

The school nurses perceived that they had an increased workload in implementing the HPV vaccinations and this was also reported in studies from the United Kingdom [10,11,21]. The available financial support had not been used to a great extent to cover the extra expenses for the vaccinations, and therefore it was not surprising that nurses reported difficulties in implementing the programme because of time constraints. Thus, it is important to identify strategies to provide direct financial support to cover the additional expenditure incurred by the vaccinations, thereby ensuring the continued success of the HPV vaccination programme.

The professional role of school nurses includes preserving and promoting health equality [9,11]. This might have been reflected in the finding that the majority agreed that boys should also be vaccinated. Other childhood vaccinations are offered to both boys and girls, so the exclusion of boys may be viewed as an ethical dilemma, which was also noted by Malmqvist et al. [22,23]. Another consideration was the attitude to the age of girls when they received the vaccination, where one-third of nurses believed that girls should be slightly older because it would have been easier to provide them with appropriate information about HPV. A majority of the school nurses also believed that parents who did not consent should be offered the option of having their daughter vaccinated at a later time. This was consistent with our previous findings about parents not consenting to vaccination [17]. Nevertheless, there is an ethical dilemma of the public health benefit of mass immunisation *versus* individual autonomy [24]. Even if it would be beneficial for an individual family to be offered vaccination at a later time, this could affect the vaccination programme and eventually lead to lower vaccination coverage. In addition, a delay in vaccination might lead to a decreased health benefit for the individual. Therefore, it must be emphasised to school nurses that the reason for vaccinating at such a young age is to give the best possible protection against HPV. One way to overcome this is by handling HPV vaccinations like all other childhood vaccinations, such as measles-mumps-rubella (MMR) vaccination. Parents who decline the MMR vaccine are contacted by the school nurse, and are offered a second chance to vaccinate. This contact with the school nurse could also be an opportunity for parents to ask additional questions about HPV and the HPV vaccine. However, it is important to emphasise that the vaccine is prophylactic, and parents should be informed that it needs to be received before possible exposure to HPV, i.e., before sexual activity commences.

### Strengths and limitations

A major advantage of this study was the high response rate. School nurses from all 20 counties in Sweden participated, and also from both private and public schools, so the results may be generalised to the whole population of school nurses involved in the HPV vaccination programme in Sweden. One caveat is that we could not be sure that school nurses from all of the 290 municipalities in Sweden participated in the survey. The use of a web-based survey allowed many nurses to participate and to respond within a short period. A possible limitation is that we could not prove that only school nurses answered the questionnaires. The national official web page was directed exclusively to school nurses, but personal identification was not required before completing the questionnaire. To overcome this limitation, we checked all the demographic data to ensure that no school nurses had responded more than once. Another possible limitation is the lack of information about non-responders.

It would have been interesting to investigate whether the attitudes of the school nurses correlated with the coverage rate, but this was not feasible as the data were not available at the time of preparation of the manuscript. Since January 2013, it has been mandatory to report vaccinations within the national childhood vaccination programme to the National Vaccination Registry in Sweden.

## Conclusions

The majority of the school nurses were in favour of the school-based HPV vaccination programme, despite the increased workload that it incurred. There were an association between received education and perceived knowledge about HPV and a positive attitude to the vaccination programme. School nurses need adequate resources so that they can provide information to parents and answer their questions and concerns. HPV vaccination is a complex and time-consuming task, which demands knowledge, skills, time and, ultimately, financial support.

## Abbreviations

CI: Confidence interval; HCP: Health care professional; HPV: Human papillomavirus; MMR: Measles-mumps-rubella; OR: Odds ratio; SD: Standard deviations.

## Competing interest

The authors declare that they have no competing interests.

## Authors’ contributions

All authors are responsible for the reported research. All authors participated in study concept and design; analysis and interpretation of the data; and drafting or revising of the manuscript. MG composed the initial manuscript, MG and AR revised it, and MG submitted the final manuscript. Statistical analyses were initially performed by AR and CS. All authors have approved the final manuscript.

## Pre-publication history

The pre-publication history for this paper can be accessed here:

http://www.biomedcentral.com/1471-2458/14/540/prepub
